# BRD4 facilitates osteogenic differentiation of human bone marrow mesenchymal stem cells through WNT4/NF-κB pathway

**DOI:** 10.1186/s13018-023-04335-x

**Published:** 2023-11-18

**Authors:** Tao Ning, Huihui Guo, Mingming Ma, Zhengang Zha

**Affiliations:** 1 Department of Bone and Joint Surgery, The First Affiliated Hospital of Jinan University, Guangzhou City, 510630 Guangdong Province People’s Republic of China; 2 Department of Orthopedics, Fuyang People’s Hospital, No.501 Sanqing Road, Fuyang City, 236000 Anhui Province People’s Republic of China

**Keywords:** Osteoporosis, hBMSCS, BRD4, WNT4, NF-κB, Glycolysis

## Abstract

**Background:**

Human bone marrow mesenchymal stem cells (hBMSCs) are a major source of osteoblast precursor cells and are directly involved in osteoporosis (OP) progression. Bromodomain-containing protein 4 (BRD4) is an important regulator for osteogenic differentiation. Therefore, its role and mechanism in osteogenic differentiation process deserve further investigation.

**Methods:**

hBMSCs osteogenic differentiation was evaluated by flow cytometry, alkaline phosphatase assay and alizarin red staining. Western blot was used to test osteogenic differentiation-related proteins, BRD4 protein, WNT family members-4 (WNT4)/NF-κB-related proteins, and glycolysis-related proteins. Metabolomics techniques were used to detect metabolite changes and metabolic pathways. BRD4 and WNT4 mRNA levels were determined using quantitative real-time PCR. Dual-luciferase reporter assay and chromatin immunoprecipitation assay were performed to detect BRD4 and WNT4 interaction. Glycolysis ability was assessed by testing glucose uptake, lactic acid production, and ATP levels.

**Results:**

After successful induction of osteogenic differentiation, the expression of BRD4 was increased significantly. BRD4 knockdown inhibited hBMSCs osteogenic differentiation. Metabolomics analysis showed that BRD4 expression was related to glucose metabolism in osteogenic differentiation. Moreover, BRD4 could directly bind to the promoter of the WNT4 gene. Further experiments confirmed that recombinant WNT4 reversed the inhibition effect of BRD4 knockdown on glycolysis, and NF-κB inhibitors (Bardoxolone Methyl) overturned the suppressive effect of BRD4 knockdown on hBMSCs osteogenic differentiation.

**Conclusion:**

BRD4 promoted hBMSCs osteogenic differentiation by inhibiting NF-κB pathway via enhancing WNT4 expression.

**Supplementary Information:**

The online version contains supplementary material available at 10.1186/s13018-023-04335-x.

## Introduction

Osteoporosis (OP) was a metabolic disease leading to increased risk of bone fragility and fracture [1, 2]. OP is characterized by decreased bone mineral density and bone microarchitecture deterioration [3]. The life quality of OP patients is diminished due to restricted mobility and chronic disability [4, 5]. According to statistics, the prevalence of OP among women aged 40 years or older is 20.6% in China [6]. Bone metabolism was a complex process, and its imbalance can lead to bone loss and changes in bone structure [7]. Metabolomics technology provides advantages for the research in the field of bone research, so the use of metabolomics technology to detect metabolites in bone metabolism can provide references for the treatment of OP [8]. The proliferation and differentiation of human bone marrow mesenchymal stem cells (hBMSCs) are closely related to the process of bone metabolism [9]. hBMSCs are mainly located in the bone marrow cavity and were multipotent stem cells with the potential for self-renewal and multidirectional differentiation (adipocytes, chondrocytes, myoblasts, and osteoblasts) [10, 11]. Therefore, promoting osteogenic differentiation of hBMSCs is of great clinical importance for OP treatment [12].

Bromodomain-containing protein 4 (BRD4), a member of the bromodomain extra-terminal protein family, participates in regulating osteogenesis, chondrogenesis and adipogenesis [13, 14]. It has been shown that BRD4 is enriched in osteoblast-specific enhancers during hBMSCs osteogenic differentiation [15]. Furthermore, BRD4 inhibitors can remedy glucocorticoid-induced OP [16]. Nowadays, BRD4 has emerged as a promising target for treating orthopedic-related disorders [17].

WNT family members-4 (WNT4) belongs to a conserved family of WNT proteins that play important roles in cell development and differentiation [18]. Increasing evidence reveals that the WNT4-mediated β-catenin pathway plays an active role in promoting hBMSCs osteogenic differentiation [19, 20]. Previous studies have indicated that WNT4 inhibits the activity of NF-κB pathway to regulate skeletal aging and new bone formation [21, 22]. In this, we found that BRD4 can promote WNT4 expression, but whether BRD4 affects osteogenic differentiation by modulating the WNT4/NF-κB pathway is unclear.

This study aimed to reveal BRD4 roles and mechanisms in osteogenic differentiation. Based on the above, we speculated that BRD4 regulated the WNT4/NF-kB pathway to mediate osteogenic differentiation.

## Methods

### hBMSCs osteogenic differentiation induction

hBMSCs were isolated from the posterior iliac crest of 6 healthy adult donors. This study was approved by the Ethics Committee at the First Affiliated Hospital of Jinan University and written consent was obtained from the participants. hBMSCs were cultured at 37 °C and 5% CO_2_ in α-MEM containing 10% FBS and 1% penicillin/streptomycin (all from HyClone, Logan, UT, USA). For osteogenic differentiation, hBMSCs were cultured in medium containing 0.1 μM dexamethasone, 0.2 mM ascorbic acid and 10 mM β-glycerophosphate (all from Sigma-Aldrich, St. Louis, MO, USA) for 21 days. Western blot of RUNX family transcription factor 2 (RUNX2), osteocalcin (OCN), bone sialoprotein (BSP) and alkaline phosphatase (ALP) were performed on the 7th day of osteogenic differentiation.

### Cell treatment and transfection

BRD4 lentiviral short hairpin RNA (shBRD4), pcDNA overexpression vector (pcDNA-BRD4) and negative controls (shNC or Vector) (GenePharma, Shanghai, China) were transfected into hBMSCs using Lipofectamine 3000 reagent (Invitrogen, Carlsbad, CA, USA). For exploring the effect of WNT4 and NF-κB pathway on BRD4-mediated osteogenic differentiation, hBMSCs were treated with 200 ng/mL human recombinant WNT4 (rWNT4) and 1 μM NF-κB inhibitors (Bardoxolone Methyl) for 24 h. After transfection or treatment, hBMSCs were induced osteogenic differentiation.

### Flow cytometry

hBMSCs in passage 0 (P0) and passage 3 (P3) were collected and incubated with cell surface antigens containing anti-CD29 (ab218273, Abcam, Cambridge, MA, USA), anti-CD34 (ab187284, Abcam), anti-CD44 (ab23396, Abcam), anti-CD90 (ab25322, Abcam), and anti-CD45 (ab27287, Abcam). Data were analyzed by FACSCalibur flow cytometer.

### Quantitative real-time PCR (qRT-PCR)

Total RNAs extracted by TRIzol reagent (Invitrogen) were used for cDNA synthesis using cDNA Synthesis Kit (Takara, Tokyo, Japan). SYBR Green (Takara) was mixed with cDNA and specific primers for PCR amplification. Relative expression was normalized by β-actin and calculated using 2^−ΔΔCT^ method. The sequences were as below: BRD4, F 5′-ACCTCCAACCCTAACAAGCC-3′, R 5′-TTTCCATAGTGTCTTGAGCACC-3′; WNT4, F 5′-AGGAGGAGACGTGCGAGAAA-3′, R 5′-CGAGTCCATGACTTCCAGGT-3′; β-actin, F 5′-CTCCATCCTGGCCTCGCTGT-3′, R 5′-GCTGTCACCTTCACCGTTCC-3′.

### Alkaline phosphatase (ALP) activity assay

After induction for 7 days, quantitative ALP staining was performed according to the instructions of ALP Assay Kit (Beyotime, Shanghai, China). In brief, hBMSCs lysate was incubated with buffer and pNPP substrate. After termination of the reaction, absorbance was measured at 405 nm to calculate ALP activity of hBMSCs.

### Alizarin red staining (ARS)

After induction for 21 days, hBMSCs were washed with phosphate-buffered saline (PBS) twice, fixed in 4% paraformaldehyde for 10 min and stained with 2% alizarin red staining (pH 4.1) for 30 min. Mineralized deposit were observed and captured under microscope.

### Western blot analysis

Total proteins were extracted using RIPA lysis buffer (Beyotime), separated by SDS-PAGE gel and transferred onto PVDF membranes. Membrane was incubated with anti-RUNX2 (ab76956, 1:1000, Abcam), anti-OCN (ab93876, 1:100, Abcam), anti-BSP (sc-73630, 1:1000, Santa Cruz, Dallas, TX, USA), anti-ALP (ab229126, 1:1000, Abcam), anti-BRD4 (ab128874, 1:1000, Abcam), anti-WNT4 (ab262696, 1:1000, Abcam), anti-IκBα (sc-1643, 1:1000, Santa Cruz), anti-p-IκBα (sc-8404, 1:1000, Santa Cruz), anti-p65 (ab16502, 1:1000, Abcam), anti-p-p65 (ab76302, 1:1000, Abcam), anti-HK2 (ab209847, 1:1000, Abcam), anti-LDHA (ab52488, 1:5000, Abcam), anti-PKM2 (ab85555, 1:1000, Abcam), anti-PFK1 (sc-166722, 1:1000, Santa Cruz) and anti-β-actin (ab8227, 1:1000, Abcam). After incubated with secondary antibody (ab205718 or ab205719, 1:50,000, Abcam), the signals were exposed using ECL reagent (Beyotime).

### Targeted metabolomics

Osteoblast precursor cells were treated with DMSO and BRD4 inhibitors (JQ1). After that, cells were treated with methanol–acetonitrile aqueous solution (2:2:1, v/v) followed by vortex for 60 s and ultrasound at low temperature for 30 min. Then, cells were placed at − 20 °C for 1 h to precipitate protein, centrifuged at 4 °C 14000rcf for 20 min, and the supernatant was collected for freeze-drying. The samples were subjected for targeted metabolomics analysis using an Agilent 1290 Infinity LC system with a 5500 QTRAP mass spectrometer (AB SCIEX). The MetaboAnalyst software was used for performing PCA, OPLS-DA and Volcano plot [23].

### Chromatin immunoprecipitation (ChIP) assay

According to the instructions of the EZ-ChIP™ kit (Millipore, Billerica, MA, USA), hBMSCs were incubated with formaldehyde to obtain DNA–protein crosslinks. After sonication to break DNA, samples were immunoprecipitated with anti-BRD4 (ab243862, Abcam) or anti-IgG (ab133470, Abcam). Finally, the precipitated DNA was subjected to qRT-PCR.

### Dual-luciferase reporter assay

The WNT4 promoter regions containing BRD4 binding sites were introduced into the pGL3-basic vector. After transfected with pGL3-basic vector and BRD4 overexpression vector or negative control (vector) into HEK-293 T (ATCC), the luciferase activity was analyzed by Dual-Luciferase Reporter Assay System (Promega, Madison, WI, USA).

### Statistical analysis

Data were expressed as mean ± SD. Statistical analyzes were performed using Student’s *t*-test or ANOVA in GraphPad Prism 7 software. Statistical significance was set as *P* < 0.05.

## Results

### Isolation and identification of hBMSCs

**Fig. 1:**
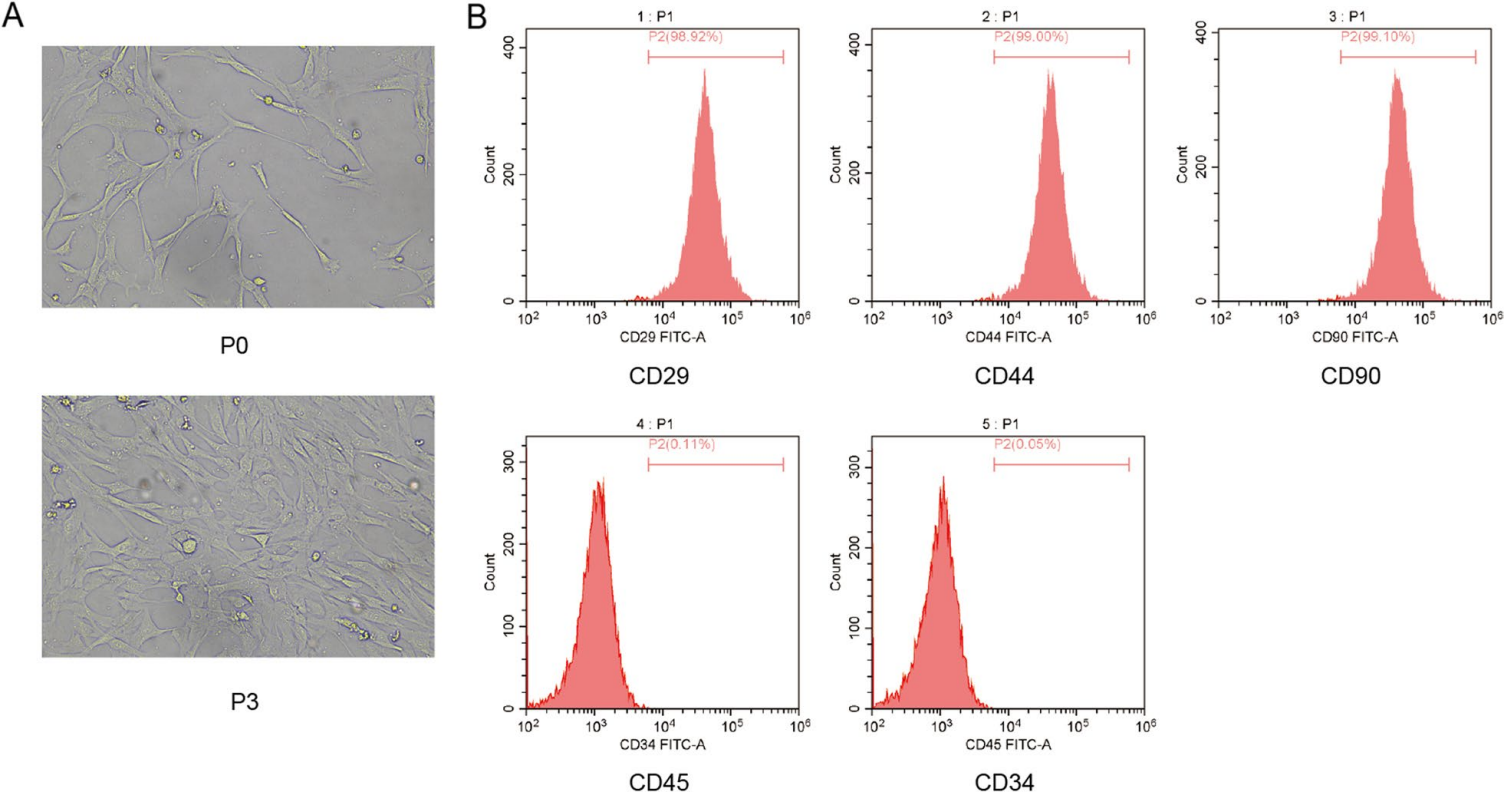
Isolation and identification of hBMSCs. hBMSCs were cultured in osteogenic differentiation medium. **A** Microscopic observation of cell morphology. **B** Identification of surface antigen molecules (CD29, CD44, CD90, CD45, CD34) on hBMSCs by flow cytometry

First, we observed the morphology of hBMSCs, which showed that hBMSCs were spindle-shaped, with fewer P0-generation cells adhered to the wall; whereas P3-generation cells were heavily fused (Fig. 1A). By further analysis, we found that the surface antigen molecules CD29, CD44 and CD90 were positively expressed, while CD34 and CD45 were negatively expressed (Fig. 1B). These results indicated that the isolated hBMSCs were successful.

### BRD4 expression was correlated with hBMSCs osteogenic differentiation

**Fig. 2:**
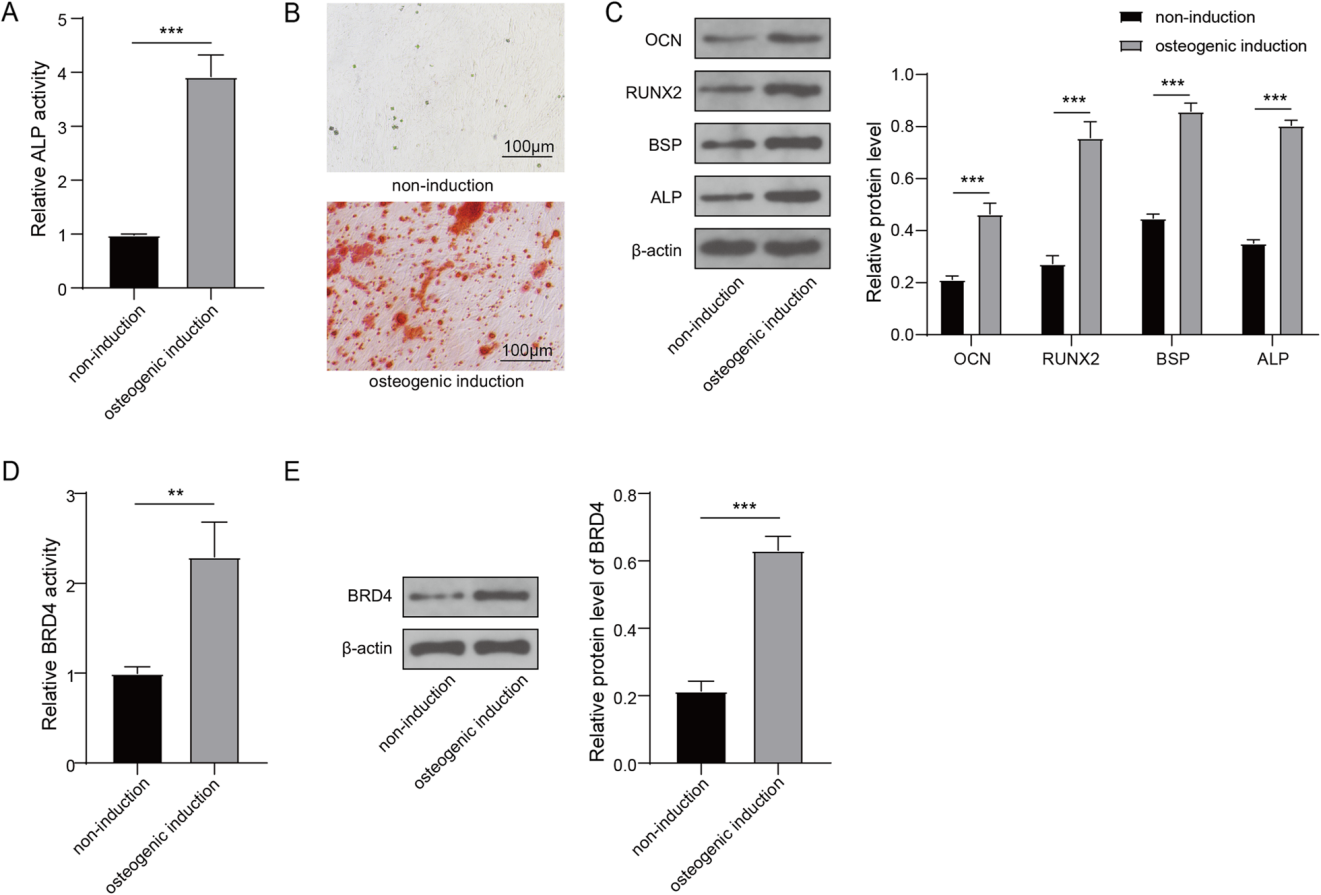
BRD4 expression in osteogenic differentiation of hBMSCs. hBMSCs were induced with or without osteogenic differentiation. **A** The ALP activity was detected by ALP Assay Kit. **B** ARS was used to detect mineralized deposit. **C** The protein expression levels of OCN, RUNX2, BSP and ALP were determined using Western blot analysis. **D** Detection of BRD4 mRNA expression by qRT-PCR. **E** Detection of BRD4 protein expression by Western blot. *n* = 3; **P < 0.01, ***P < 0.001

To investigate the success of induced osteogenic differentiation in hBMSCs, we carried out relevant tests. ALP activity was remarkably higher in osteogenic induction group of hBMSCs than in the non-induction group (Fig. 2A). ARS indicated that hBMSCs produced more mineralized deposit after osteogenic induction (Fig. 2B). Western blot results revealed that the protein expression levels of OCN, RUNX2, BSP and ALP were also markedly enhanced in hBMSCs after induction of osteogenic differentiation (Fig. 2C). We also examined the mRNA and protein expression levels of BRD4 and found that BRD4 expression was observably upregulated in hBMSCs after osteogenic differentiation (Fig. 2D, E). These data showed that BRD4 expression was related to hBMSCs osteogenic differentiation.

### BRD4 silencing inhibited osteogenic differentiation of hBMSCs

**Fig. 3:**
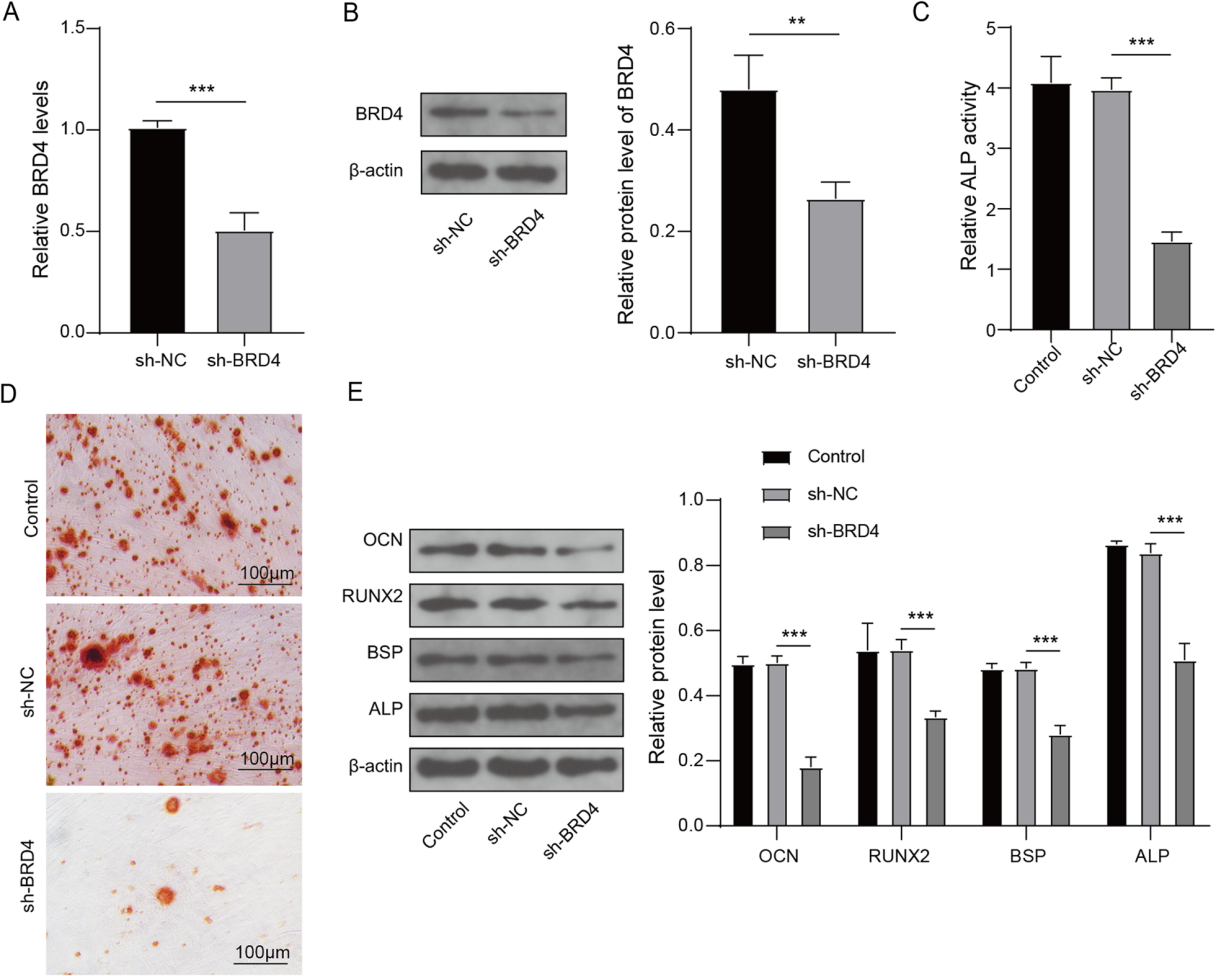
Effect of shBRD4 on osteogenic differentiation of hBMSCs. hBMSCs were transfected with shNC or shBRD4. **A** Detection of BRD4 mRNA expreesion by qRT-PCR. **B** Detection of BRD4 protein expression using Western blot analysis. **C** The ALP activity was measured using ALP Assay Kit. **D** ARS was performed for assessing mineralized deposit. **E** The protein expression levels of OCN, RUNX2, BSP and ALP were determined using Western blot. *n* = 3; **P < 0.01, ***P < 0.001

To explore the role of BRD4 on hBMSCs osteogenic differentiation, shBRD4 was transfected into hBMSCs to silence BRD4. QRT-PCR and Western blot analysis were used to assess the transfection efficiency of shBRD4, and the results indicated that BRD4 mRNA and protein expression levels were significantly reduced after shBRD4 transfection (Fig. 3A, B), which proved that transfection was effective. ALP activity detection and ARS results showed that BRD4 knockdown suppressed ALP activity (Fig. 3C) and reduced mineralized deposit in hBMSCs after osteogenic differentiation (Fig. 3D). Besides, the protein levels of osteogenic differentiation-related markers (OCN, RUNX2, BSP and ALP) were obviously decreased by BRD4 knockdown (Fig. 3E). All results revealed that BRD4 knockdown suppressed the osteogenic differentiation of hBMSCs, confirming that BRD4 might promote hBMSCs osteogenic differentiation.

### BRD4 expression was associated with glucose metabolism in osteogenic differentiation

**Fig. 4:**
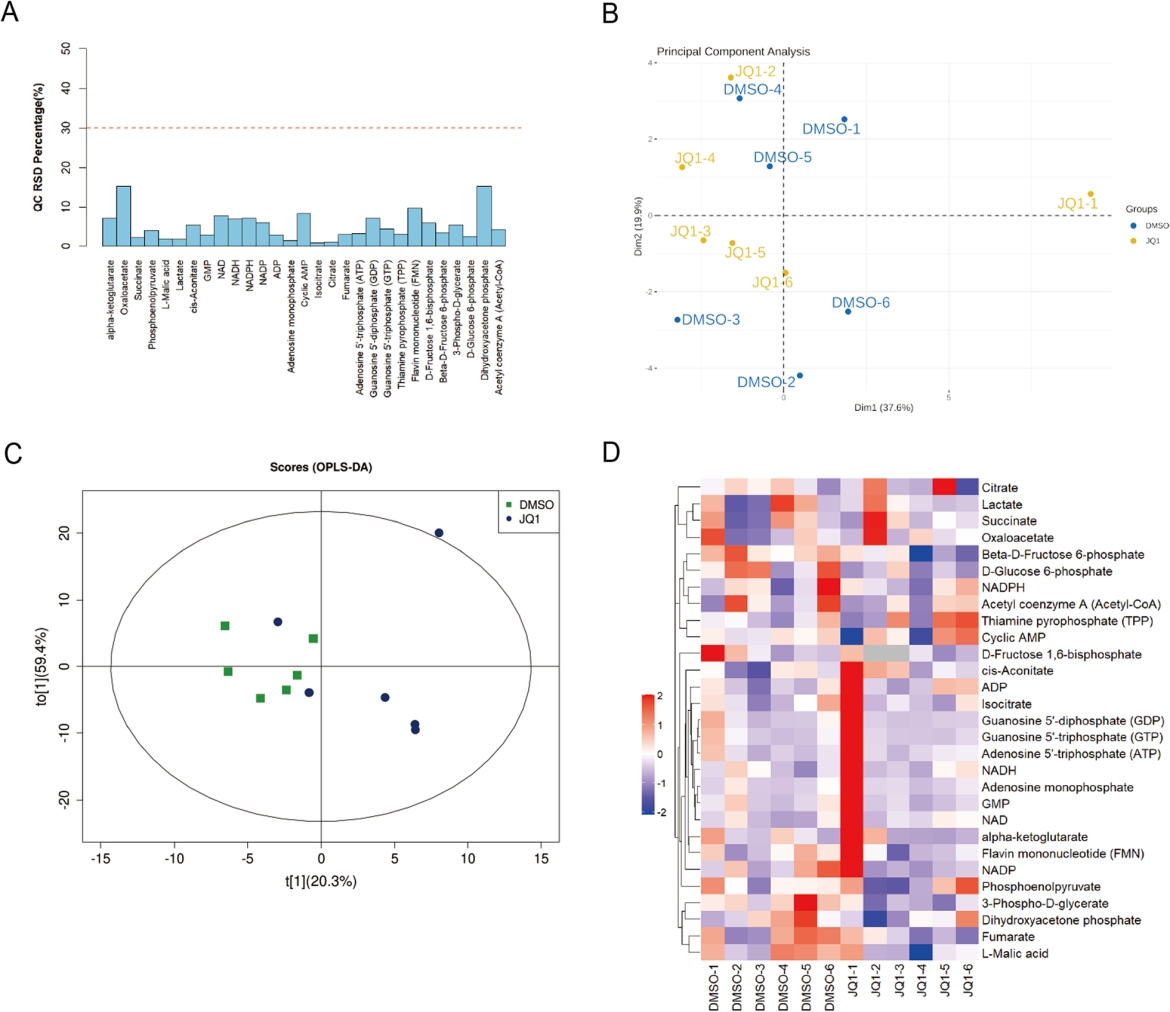
Metabolomics analyzed the relationship of BRD4 inhibition and glucose metabolism. Osteogenic precursor cells were treated with DMSO and BRD4 inhibitors (JQ1). **A** Analyze QC samples to assess analytical precision. **B, C** Composition of endogenous metabolites in osteogenic precursor cells by PCA analysis and OPLS-DA analysis. **D** The results of the screening of the different metabolites are shown in heat maps. *n* = 6

To investigate whether the inhibition of BRD4 during osteogenic differentiation is related to glucose metabolism, osteogenic precursor cells were treated with DMSO and BRD4 inhibitors (JQ1). Before data analysis, inspection of Quality Control (QC) samples was performed as a typical procedure for the assessment of the analytical precision to ensure that any variations in the metabolic content of treated vs. control samples were due to intervention. Only compounds with RSD less than 30% were further investigated (Fig. 4A). Using metabolomics techniques, the experiments were analyzed by PCA and OPLS-DA, and the results revealed that endogenous metabolites of osteogenic precursor cells were found to be significantly different in the JQ1-treated group compared to the DMSO control group (Fig. 4B, C). Through screening of metabolites by box plots (Additional file 1: Figs. S1–S4) and heat maps, we found that Beta-D-fructose 6-phosphate, 3-phosphate-D-glycerate and D-glucose 6-phosphate, 3 compounds related to glucose metabolism, were significantly different in JQ1 group and control group (Fig. 4D). These data confirmed that BRD4 expression was related to glucose metabolism.

### BRD4 transcriptionally upregulated WNT4 expression

**Fig. 5:**
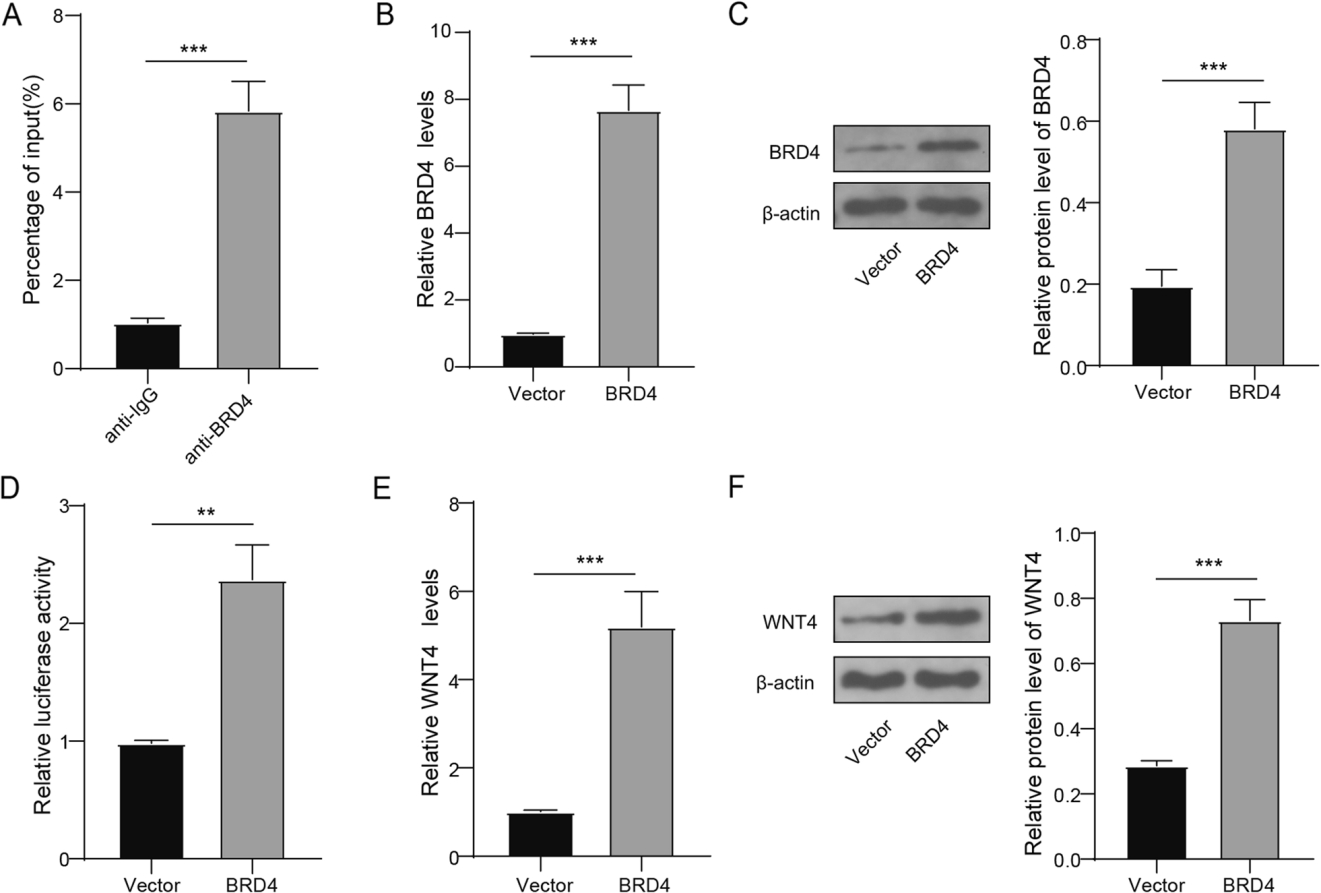
BRD4 regulated the expression of WNT4. **A** The interaction between BRD4 and WNT4 was detected by ChIP. **B–F** BRD4 overexpression vector or vector was transfected into hBMSCs. **B** Detection for mRNA expression of BRD4 using qRT-PCR. **C** Western blot analysis was used to examine the protein expression of BRD4. **D** The binding of BRD4 to WNT4 promoter was detected using dual-luciferase reporter assay. **E** Detection for mRNA expression of WNT4 using qRT-PCR. **F** The protein expression of WNT4 was detected by Western blot. *n* = 3; **P < 0.01, ***P < 0.001

To verify the interaction between BRD4 and WNT4, we preformed experiment analysis. ChIP assay confirmed that BRD4 could directly bind to the promoter regions of the WNT4 gene (Fig. 5A). In addition, we constructed BRD4 overexpression vectors for upregulating the mRNA and protein levels of BRD4 in hBMSCs (Fig. 5B-C). Dual-luciferase reporter assays showed that the binding of BRD4 to WNT4 was increased after BRD4 overexpression in hBMSCs (Fig. 5D). Further analysis indicated that WNT4 mRNA and protein levels were promoted by BRD4 overexpression (Fig. 5E-F). The above noted that BRD4 enhanced WNT4 expression.

### BRD4 affected osteogenic differentiation of hBMSCs by promoting glycolysis through regulating WNT4/NF-κB pathway

**Fig. 6:**
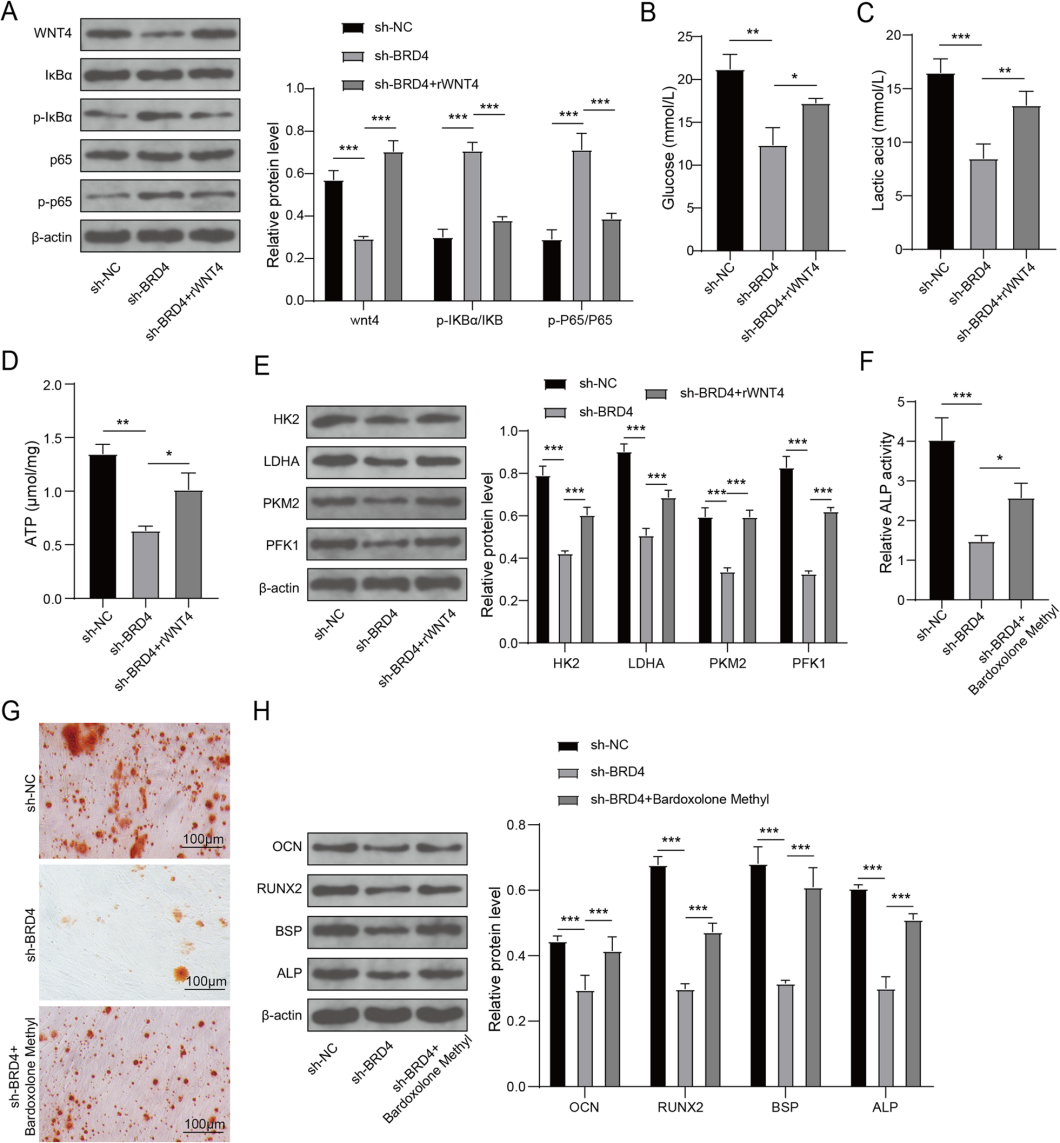
Effect of shBRD4, rWNT4 and NF-κB inhibitors on glycolysis and osteogenic differentiation of hBMSCs. hBMSCs were transfected with shNC or shBRD4 and treated with rWNT4 or Bardoxolone Methyl followed by induced osteogenic differentiation. **A** Detection of WNT4, IκBα, p-IκBα, p65 and p-p65 expression using Western blot. **B-D** Glycolysis was detected by measuring glucose uptake, lactic acid production and ATP levels. **E** Western blot assay was used to examine the expression levels of key glycolytic enzymes (HK2, LDHA, PKM2 and PFK1). **F** ALP activity was tested by ALP Assay Kit. **G** ARS was utilized to assess mineralized deposit. **H** The protein expression levels of OCN, RUNX2, BSP and ALP were determined using Western blot analysis. *n* = 3; *P < 0.05, **P < 0.0, ***P < 0.001

To test whether BRD4 affects the osteogenic differentiation of hBMSCs by regulating the WNT4/NF-κB pathway, hBMSCs were transfected with shBRD4 and treated with rWNT4 followed by inducting osteogenic differentiation. The inhibitory effect of BDR4 knockdown on WNT4 expression, as well as the promotive effect on p-IκBα/IκBα and p-p65/p65 expression, could be reversed by rWNT4 (Fig. 6A). Furthermore, BRD4 knockdown clearly inhibited cellular glucose uptake, lactic acid production and ATP levels, which were significantly increased by rWNT4 treatment (Fig. 6B–D). Western blot assay analyzed the expression levels of key glycolytic enzymes (HK2, LDHA, PKM2 and PFK1), and the results revealed that rWNT4 reversed the decreasing effect of shBRD4 on the above glycolysis-related proteins (Fig. 6E). In addition, hBMSCs were transfected with shBRD4 and treated with NF-κB inhibitors (Bardoxolone Methyl). After inducing osteogenic differentiation, we examined ALP activity, mineralized deposit and osteogenic differentiation marker proteins. As shown in Fig. 6F–H, NF-κB inhibitors reversed the suppression effect of BRD4 knockdown on ALP activity, mineralized deposit and the protein levels of OCN, RUNX2, BSP and ALP. These data indicated that BRD4 regulated the WNT4/NF-kB pathway to mediate osteogenic differentiation.

## Discussion

Many studies have found that hBMSCs are indispensable for therapeutic play in skeletal diseases. For example, Shang et al*.* found that hBMSCs osteogenic differentiation is important for regeneration of bone defects [24]. Zhang et al*.* discovered that hBMSCs are a major source of osteoblast precursor cells and are directly involved in the progression of OP [25]. Hence, it is important to identify the molecular mechanisms affecting osteogenic differentiation of hBMSCs for the treatment of OP.

The role and mechanism of BRD4 in the osteogenic differentiation of hBMSCs has been investigated in many studies. Wang et al*.* found that BRD4 inhibitors could diminish the hBMSCs osteogenic differentiation [26], and Paradise et al*.* obtained the same result [27]. Our study showed that BRD4 expression was increased after hBMSCs osteogenic differentiation, and its knockdown significantly inhibited the ability of hBMSCs osteogenic differentiation, which was consistent with previous studies [26, 27]. This evidence suggested that BRD4 might be a potential target for OP treatment.

Over the years, metabolomics has emerged as a technique for the discovery of active drivers of biological processes [28–30]. It detects the changes in multiple metabolites during environmental exposures in a high-throughput format and is strongly associated with pathological phenotypes, especially in multifunctional diseases such as OP [8, 31]. Importantly, the application of metabolomics has facilitated the study of OP, and further exploration of the mechanism and behavior of osteoblasts from the metabolic perspective can provide new ideas for OP treatment [32]. In this, we used metabolomics to identify that BRD4 expression was associated with 3 compounds involved in glucose metabolism in osteoblast precursor cells.

WNT signaling plays an active role in osteogenic differentiation [33]. WNT4, a promoter of osteogenic differentiation of hBMSCs, is involved in regulating bone homeostasis and bone formation in OP [18], and has potential application value in improving bone regeneration and repairing craniofacial defect [34]. Xu et al*.* found that WNT4 promoted bone regeneration by inhibiting IKK-NF-κB signaling [35]. Yu et al*.* reported that WNT4 attenuated OP progression by inhibiting nuclear factor NF-κB [22]. Previous study showed that BRD4 aggravated neuroinflammatory response via inhibiting HMGB-1 expression [36]. Moreover, BRD4 could promote PLK1 expression through P300/H3K27ce, thereby enhancing hepatic stellate cells activation and hepatic fibrosis [37]. Our experimental results revealed that BRD4 could directly bind to the promoter of the WNT4 gene to promote WNT4 expression. Also, BRD4 knockdown reduced WNT4 expression to repress glycolysis of hBMSCs. Further analyzes identified that NF-κB inhibitors reversed the inhibitory effect of BRD4 knockdown on hBMSCs osteogenic differentiation, confirming BRD4 regulated WNT4/NF-κB pathway to mediate osteogenic differentiation.

## Conclusions

On the basis of the above studies, we pointed out that BRD4 enhanced WNT4 expression to inhibit the activity of NF-κB pathway, thereby enhancing glycolysis to aggravate hBMSCs osteogenic differentiation. These findings have far-reaching implications as they provide potential targets for OP treatment.

### Supplementary Information

**Additional file 1: Figs. S1–S4.** Metabolomics analyzing. The screening of the different metabolites in osteogenic precursor cells treated with DMSO and JQ1 are shown in box plots. *n* = 6.

## Data Availability

The datasets used and/or analyzed during the current study are available from the corresponding author on reasonable request.

## Abbreviations

hBMSCs: Human bone marrow mesenchymal stem cells
OP: Osteoporosis
BRD4: Bromodomain-containing protein 4
ALP: Alkaline phosphatase
qRT-PCR: Quantitative real-time PCR
ARS: Alizarin red staining
WNT4: WNT family members-4
