# Supplementary figures and images for: BRD4 facilitates osteogenic differentiation of human bone marrow mesenchymal stem cells through WNT4/NF-κB pathway
Source: J Orthop Surg Res. 2023 Nov 18;18:876. doi: 10.1186/s13018-023-04335-x (PMC10656925; doi:10.1186/s13018-023-04335-x)

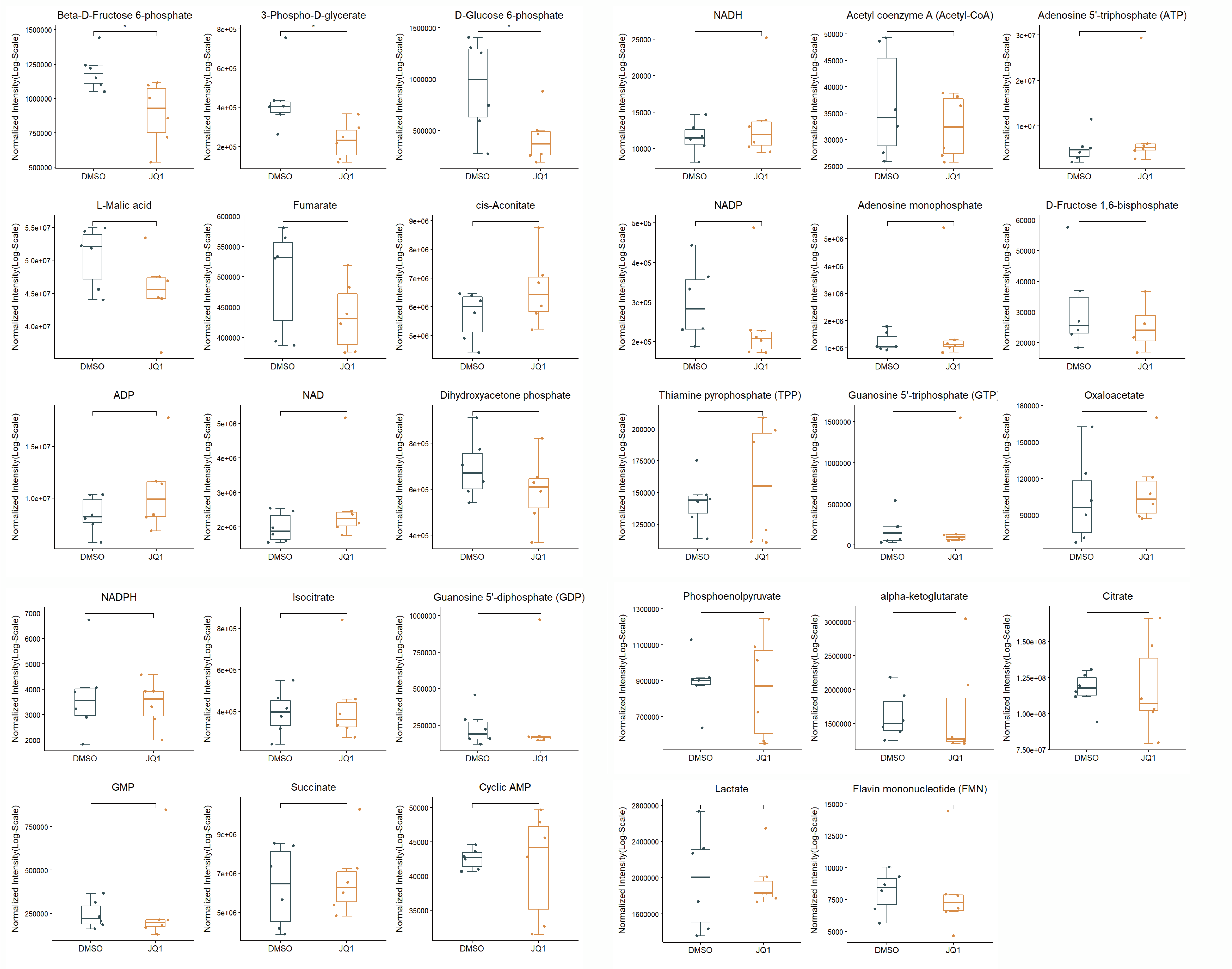

Supplement: Supplementary file 1 — Additional file 1: Figs. S1–S4. Metabolomics analyzing. The screening of the different metabolites in osteogenic precursor cells treated with DMSO and JQ1 are shown in box plots. n = 6. [file 13018_2023_4335_MOESM1_ESM.tif]
